# Validation and cultural adaptation of the Integrated Palliative care Outcome Scale (IPOS) for the Portuguese population

**DOI:** 10.1186/s12904-020-00685-z

**Published:** 2020-11-24

**Authors:** Bárbara Antunes, Pedro Lopes Ferreira

**Affiliations:** 1 Primary Care Unit, Department of Public Health and Primary Care, University of Cambridge, Cambridge, UK; 2 Centre for Health Studies and Research, University of Coimbra, Coimbra, Portugal; 3 Faculty of Economics, University of Coimbra, Coimbra, Portugal

**Keywords:** Patient-centred outcome measures, Palliative care, Outcome measurement, Palliative care outcome scale, Validation

## Abstract

**Background:**

To culturally adapt and validate the Integrated Palliative care Outcome Scale to European Portuguese.

**Methods:**

Multi-centred observational study with 2 assessment points. Data were collected in nine centres using consecutive sampling. All patients were screened for eligibility. Inclusion criteria: ≥18 years, mentally fit to give consent, diagnosed with an incurable, potentially life-threatening illness, read, write and understand Portuguese. Translation and back translation with independent native speakers blind to the original measure created a Portuguese version, which was culturally adapted using cognitive interviews. For psychometric testing, the COSMIN checklist was followed. Reliability and content validity were assessed for patient and staff versions. Construct and criterion validity were tested for patient version.

**Results:**

1703 individuals were screened between July 1st 2015 and February 2016, 135 (7.9%) were included. Mean age was 66.8 years (SD 12.7), 58 (43%) were female. Most patients (109; 80.7%) had a cancer diagnosis. Cronbach’s alpha showed good internal consistency, 0.657 for patient, 0.705 for staff versions. Intraclass correlation coefficient testing reproducibility revealed very good reliability, 0.794–0.950 for patient and 0.456–0.925 for staff versions. There was good content validity and significant results for construct validity. Physical symptoms were better detected by females. IPOS could discriminate: practical issues in different places of care, based on cancer diagnosis, physical and emotional symptoms based on life expectancy both for patient and professional dimensions, physical and emotional symptoms based on phase of illness, for professional dimensions, and physical symptoms from the patients’ viewpoint.

**Conclusions:**

The Portuguese IPOS is a reliable and valid measure.

**Supplementary Information:**

The online version contains supplementary material available at 10.1186/s12904-020-00685-z.

## Background

The Integrated Palliative care Outcome Scale (IPOS) [1] is a patient-centred outcome measure resulting from the merge of two previously existing measures: the Palliative care Outcome Scale and the Palliative care Outcome Scale-Symptoms [2]. IPOS was developed at the request of several clinicians wanting a more user-friendly measure for clinical practice. Two versions were developed, one to be self-reported by patients, and a staff-proxy-reported to be filled by healthcare professionals, considering the perceptions and holistic assessment of the symptoms and other concerns the patient might have.

The aim of this study was twofold: (i) to translate, culturally adapt and validate the original English IPOS into Portuguese, and (ii) to compare the results obtained by the two versions of IPOS.

## Methods

### Linguistic and cultural adaptation

We followed the internationally defined methodology for the linguistic and cultural adaptation for the validation of outcome measures proposed by the COnsensus-based Standards for the selection of health Measurement INstruments (COSMIN) checklist [3] and the sequential approach for the translation [4].

After seeking and getting consent from the authors of the original IPOS measure, two independent bilingual native Portuguese speaking translators, one clinical and one non-clinical, both blind to the original English IPOS for patients, created two Portuguese versions. Next, two other native Portuguese speaking independent reviewers not blind to the original IPOS developed a consensus Portuguese version. This was then sent to two other independent native English-speaking translators, also blind to the original English IPOS, who back translated it into English. Comparing this back translated version with the original one, the same reviewers developed a second Portuguese consensus version.

Then, three clinical revisions were performed by one specialist palliative care doctor, one specialist palliative care nurse and one non-clinical researcher in palliative care – all native Portuguese. These experts were asked to look at both versions and comment for each question. Three possible outcomes might arise: (1) question was correctly written and no change was proposed, (2) question was incorrectly phrased and an alternative question was proposed; or (3) question was correctly written but an alternative would better. Based on the patient version, a final clinical review of the staff version of IPOS was also performed by the same experts.

Next, cognitive interviews were performed with 12 palliative care patients and nine healthcare professionals. Each patient had an individual interview and professionals were interviewed in two groups. The purpose of these interviews was to check acceptability by patients and staff, without ambiguity and redundancy and lack of important items. A final Portuguese version was obtained [5].

### Study design

This was a multi-centred observational study with two assessment points. Data were collected in nine centres using consecutive sampling. All patients attending the participant services were screened for eligibility. Inclusion criteria were to be 18 years or older, mentally fit to give consent, diagnosed with an incurable, potentially life-threatening illness, read, write and understand Portuguese. Exclusion criteria included patients in distress or cognitively impaired. All professionals who provided care for participant patients were eligible for this study. They filled the staff proxy IPOS independently from the corresponding patients.

A standard operating procedures manual was previously developed and distributed to all centres in the person of the facilitator/champion leading the study locally.

After checking data quality, Little’s MCAR test was implemented to verify if data were missing at random. We adopted the following criteria: rates < 1% are trivial, 1–5% are manageable, 5–15% require sophisticated statistical methods to handle, and >  15% may severely impact any form of interpretation [6].

Ethical approvals were granted in accordance with the 1964 Helsinki declaration and its later amendments or comparable ethical Standard be the following Ethics Committees: S. João Hospital (no reference number), Regional Health Authorities of Centro (reference 77/2015) and of Lisbon and Tagus Valley (reference 6801/CES/2015), Vila Nova de Gaia/Espinho Hospital Centre (reference 275/2015), Lisbon Cancer Institute (reference UIC/967 n° 89/2015), S. João de Deus Institute (reference CEISJD03_15); Lisbon Medicine Academic Centre (reference 51/15), and Nordeste Health Local Unit (no reference number). Informed written (signed) consent was obtained from all participants (patients and healthcare professionals). SPSSv22 software was used.

### Measures used

IPOS [1], the measure under study, is a brief, 19-item, multidimensional scale designed to capture core concerns in palliative care. Item 1 is an open question regarding the three main problems or worries the patient/professional had in the past week; items 2 to 9 are set on 5-point Likert scales based on descriptors, item 2 lists ten of the most common physical symptoms in a palliative population, with the possibility of an additional three symptoms (not present in the list); item 3 is about anxiety, item 4 pertains to family/friends worries, item 5 asks about depression; item 6 is feeling at peace; item 7 addresses sharing feelings with significant people; item 8 is about information needs and item 9 concerns practical problems related to their illness. In the patient version, there is an extra item asking if they had filled the questionnaire alone or with help. At the very end, there is a trigger in the form of a footnote noting that if the patient became worried about any of the issues raised by the questionnaire, they should/are advised to talk to their healthcare professional about those worries.

Each of the ten physical symptoms and all the following four emotional symptoms were linearly converted into a 0–100 scale, in which higher scores corresponded to higher severity symptoms. Additionally, items 7 to 9 were converted to a 0–100 scale representing the functionality associated to each question. An average for each symptom and functionality item was accordingly computed.

The Portuguese EuroQol questionnaire (EQ-5D-3L) [7] allows quantifying two main components of health related quality of life: a first description of health status in the form of five dimensions or domains and a numeric value associated with the perceived global health status by the individual. These components are used in cost-utility economic evaluations, after transforming the descriptive system into a unique utility score. There are five items set in a 3-point Likert scale with descriptors. The items pertain to mobility, self-care, usual activities, pain/discomfort and anxiety/depression. It also has a numeric analogue visual scale (EQ-VAS) from 0 (the worst possible health state) to 100 (the best possible health state) so that the respondent can quantify their health state in the moment when filling the questionnaire. The authors of the Portuguese version state that this measure has good accessibility, reliability and validity in measuring health [7, 8]. The obtained utility score ranges from 0 (death) to 1 (perfect health), allowing for negative values corresponding to health state perceived as worse than death.

The Portuguese translation of the European Organization for Research and Treatment of Cancer Quality of Life Questionnaire Core (EORTC QLQ-C30) [9, 10] is a 30-item questionnaire, 24 items compose nine multi-item scales, namely, five functional subscales (physical, role, cognitive, emotional, and social); a global health/QoL subscale and three symptom subscales (fatigue, pain, and nausea/vomiting). The remaining six items are single-item assessing symptoms commonly reported by cancer patients (dyspnoea, appetite loss, sleep disturbance, constipation, and diarrhoea) and one remaining item related to the perceived financial impact of cancer. All items are scored on 4-point Likert scale ranging from 1 (not at all) to 4 (very much), apart from two items of the global health/QoL subscale, which use a modified 7-anchor linear analogue scale. All scales range from 0 to 100. A high score for functional scales and global health status/QoL represents a healthy level of functioning and QoL. For each subscale, a score between 0 and 7 is considered normal, between 8 and 10 mild, between 11 and 14 moderate and between 15 and 21 severe. The authors conclude that the Portuguese EORTC QLQ C-30 has good metric properties.

The Portuguese Hospital Anxiety and Depression Scale (HADS) [11] screens for anxiety and depression states and has 14 items divided into two subscales. These are based on a 4-point Likert scale, with descriptive answers, comprised of seven items each, scored separately. The authors propose a clinical cut-off of 11 for depression and anxiety. The authors conclude that the Portuguese HADS is reliable and valid to assess depression and anxiety in different medical settings and disease populations.

‘Phase of illness’ is a conceptualization of a patient’s illness in five distinct, clinically meaningful phases—stable, unstable, deteriorating, terminal, bereavement (this last omitted in the present study) - developed in context of the Australian Case Mix Classification. It can be used as an indicator of acuity and reflects complexity within the disease trajectory [12, 13].

Demographic and clinical variables were also collected. The content in the open questions 1a, 1b and 1c was aggregated to define the most prevalent worries occurring in the previous week of completion.

### Reliability

Reliability was addressed by the intertemporal stability and the internal consistency. The intertemporal stability was tested in inpatients and outpatients with a guarantee of no clinical change, by intraclass correlation coefficient (ICC), on average, in a 1-week test-retest design. A score smaller that 0.5 is considered weak, between 0.5 and 0.75 moderate, between 0.75 and 0.9 good, and larger than 0.9 excellent [14]. The inter-rater reliability between staff members was also assessed.

Internal consistency was tested by the Cronbach’s alpha coefficient, which should have scores between 0.7 and 0.9 [15]. However, following author’s advice [1], we also lowered the lower limit 0.6 due to the multi-dimensionality and non-redundancy of IPOS [16].

The following two hypotheses were formulated:

H_1_: The Portuguese version of the IPOS shows good intertemporal stability.

H_2_: The Portuguese version of the IPOS shows good internal consistency.

### Validity

Validity was addressed by the content, construct, and criterion validity [15, 16]. The former has been tested through the clinical reviews and the cognitive debriefing interviews.

Construct validity was tested by hypotheses with known groups stratified by sociodemographic variables (age, gender, and education level) and some clinical variables (place of care, type of disease, life expectancy and phase of illness).

The following hypotheses were raised:

H_3_: IPOS can discriminate based on the sociodemographic variable age.

H_4_: IPOS can discriminate based on the sociodemographic variable gender.

H_5_: IPOS can discriminate based on the sociodemographic variable education level.

H_6_: IPOS can discriminate based on the clinical variable place of care.

H_7_: IPOS can discriminate based on the clinical variable type of disease.

H_8_: IPOS can discriminate based on the clinical variable life expectancy.

H_9_: IPOS can discriminate based on the clinical variable phase of illness.

Age and gender were not expected to influence IPOS scores. Less educated patients might have more symptoms with greater impact on quality of life. Same phenomenon was expected for unstable patients.

Normality tests were first performed. Two-sample independent t-tests were applied when normality assumed; if not, chi-squared and Mann-Whitney U tests were used.

*P* values below 0.05 are considered statistically significant.

To test the criterion validity, we used bivariate statistical analyses (Pearson’s r correlation coefficients) between the dimensions of the Portuguese version of the IPOS and other measuring instruments, namely EQ-5D-3L (index and VAS), EORTC QLQ-C30 (functional index, symptom scale, and quality of life) and HADS (anxiety and depression) items. We hypothesised that items measuring similar constructs would have higher correlations and items measuring different constructs would have a lower correlation. Correlations less than 0.3, between 0.3 and 0.5, and higher than 0.5 were defined as weak, moderate and strong, respectively [17].

The following three hypotheses were formulated:

H_10_: IPOS dimensions are correlated with similar EQ-5D-3L dimensions.

H_11_: IPOS dimensions are correlated with similar EORTC QLQ-C30 dimensions.

H_12_: IPOS dimensions are correlated with similar HADS dimensions.

We expect convergence between similar dimensions and divergence for distinct dimensions’ scores.

## Results

### Linguistic and cultural adaptation

There were grammatical and content differences in the first translation stage, regarding items/questions phrasing as well as the response categories. There were differences in the backward translation, namely verb tenses and the use of synonyms, rather than the direct translation of words. Both issues were resolved by discussion with both reviewers.

The clinical revisions flagged differences in verb tenses in three items which were discussed, and changes were made to create the final version. In relation to the cognitive interviews to determine content (face) validity, these were conducted in two palliative care services in two major hospitals in the North of Portugal. Twelve patients were individually interviewed, and nine healthcare professionals were interviewed in two groups. See Supplemental Material Table 1 for demographics, time of completion and opinions on the IPOS from all respondents.

Main changes to finalise the Portuguese IPOS were: (i) to remove numerals indicative of the scores in the different items in each response square; (ii) to alter the questionnaire instructions of the healthcare professional to clarify that the last response option should be used for “not applicable” as well as “unknown”; (iii) to alter the instructions in the patient questionnaire to avoid repetition of requesting the respondent to choose the best answer and mark it with an “x” in the corresponding square; (iv) to add space between the groups of items in page 2 as well as add the time period to which the items pertain to, namely, “During the last week”.

### Data collection

Data were missing at random (Little’s MCAR test showed Chi-Square = 2452.9; *p* = 0.213). Missing data varied between 1 and 5%, as expected in palliative populations, most questionnaire items presented a non-parametric distribution, so the imputation of the median was used to handle missing data.

### Demographic and clinical data

1703 individuals were screened in nine centres of mainland Portugal between July 1st 2015 and 20th February 2016. Among them, 1410 (82.8%) were immediately considered non eligible, mainly because they were not enrolled in palliative care, were less than 18 years of age, or could not read, write or understand Portuguese. We excluded 140 (8.2%) more individuals mainly because they were excessively suffering, or they were cognitively deteriorated. There were 18 (1.1%) eligible patients who declined participation.

A total of 135 (7.9%) patients were included and filled the questionnaires without help, most (98; 72.6%) were approached to participate in the study whilst in outpatient consultations. Table 1 presents demographic and clinical information for respondent patients.

**Table 1:** Demographic and clinical information of participating patients

		Patients	
Variable	Value	N	%
Gender	Male	77	57.0
	Female	58	43.0
Age (years)	< 65	57	42.2
	65+	78	57.8
	Min – Max	29–94	
	Mean ± standard deviation	66.8 ± 12.7	
Education	Reads and writes	5	3.7
	4 years	81	60.0
	6 years	20	14.8
	9 years	10	7.4
	10 years to college	19	14.0
Geographical Region	North	74	54.8
	Centre	25	18.5
	South	36	26.7
Area	Urban	94	69.6
	Peri-urban	31	23.0
	Rural	10	7.4
Place of care	Primary care	28	20.7
	Hospital	25	18.5
	Palliative care	82	60.7
Cancer diagnosis	Yes	109	80.7
	No	26	19.3
Life expectancy	Less than 6 months	48	35.6
	Between 6 months and 1 year	47	34.8
	More than 1 year	40	29.6
Phase of illness	Stable	64	47.4
	Unstable	28	20.7
	Deteriorating	43	31.9
Quality of Life	Mean Index	31.4	
	Mean VAS	53.7	
Anxiety (HADS)	Normal (0–7)	11	8.1
	Mild (8–10)	16	11.9
	Moderate (11–14)	66	48.9
	Severe (15–21)	42	31.1
	Mean ± standard deviation	12.7 ± 3.1	
Depression (HADS)	Normal (0–7)	17	12.6
	Mild (8–10)	63	46.7
	Moderate (11–14)	48	35.6
	Severe (15–21)	7	5.2
	Mean ± standard deviation	10.1 ± 2.6	
EORTC Quality of Life		49.7 ± 20.6	
EORTC Functional scales	Physical functioning	48.4 ± 28.7	
	Role functioning	51.5 ± 34.7	
	Emotional functioning	62.5 ± 24.2	
	Cognitive functioning	75.4 ± 23.2	
	Social functioning	66.9 ± 30.9	
EORTC Symptom scales	Fatigue	49.0 ± 25.4	
	Nausea and vomiting	8.1 ± 16.5	
	Pain	35.1 ± 29.1	
	Dyspnoea	14.1 ± 26.5	
	Insomnia	32.6 ± 34.4	
	Appetite loss	30.9 ± 36.6	
	Constipation	24.9 ± 31.7	
	Diarrhoea	9.1 ± 20.9	
	Financial impact	32.6 ± 35.8	

Most patients were older male with low literacy, mainly diagnosed with cancer, receiving specialized palliative care, and in a stable phase of their illness. However, their mean quality of life index was low, they showed high scores of anxiety and low physical and emotional functioning scores. Their main reported symptoms were fatigue and pain.

In Table 2 we present the mean scores for each IPOS item when filled by patients and assigned by healthcare professionals. By comparing, for each patient, his/her score and the one provided by the healthcare professional, we also present the results from the paired samples t-test.

**Table 2:** Descriptive statistics of IPOS items (%) - *n* = 134

		Patients	Staff	|t| (Sig.)
*Physical symptoms*	Pain	42.0	35.9	1.914 (*p* = 0.058)
	Shortness of breath	15.0	14.8	0.118 (*p* = 0.906)
	Lack of energy	42.1	45.6	1.598 (*p* = 0.112)
	Nausea	12.6	11.3	0.733 (*p* = 0.465)
	Feeling sick	8.7	6.4	1.801 (*p* = 0.074)
	Poor appetite	34.5	30.4	1.374 (*p* = 0.172)
	Constipated	28.2	19.0	3.923 (***p*** **< 0.001**)
	Wounds in mouth or mouth dry	31.4	10.6	7.450 (**p < 0.001**)
	Sleepiness	33.1	14.7	7.493 (***p*** **> 0.001**)
	Lack of mobility	39.2	41.4	1.029 (*p* = 0.305)
	Total subscale score	28.7	23.3	5.233 (**p < 0.001**)
*Emotional symptoms*	Anxiety	56.9	54.5	0.921 (*p* = 0.359)
	Family/friends worry	70.3	62.4	2.693 (***p*** **= 0.008**)
	Depression	47.3	42.0	2.103 (*p* = 0.037)
	Feeling at peace	35.8	45.0	3.151 (***p*** **= 0.002**)
	Total subscale score	52.7	50.7	1.206 (*p* = 0.230)
*Communications/* *practical issues*	Sharing feelings	62.9	48.6	4.285 (**p < 0.001**)
	Info needs	83.6	80.9	0.939 (*p* = 0.349)
	Practical problems	76.5	68.8	2.487 (***p*** **= 0.015**)
	Total subscale score	73.9	65.4	4.217 (**p < 0.001**)

P values below 0.05, considered statistically significant, are in bold letters

Emotional symptoms are more prevalent, especially those measuring family and patient anxiety, and depression. Regarding physical symptoms, the presence of weakness or lack of energy, pain, and poor mobility were the most prevalent. Vomiting, nausea and shortness of breath are the symptoms less present in these patients.

Comparing patients and professionals scores, we note lower scores among professionals regarding physical symptoms as constipation, wounds in mouth or mouth dry, and trouble in sleep, as well as the emotional symptoms, being worried about family and friends, depressed, or feeling at peace. How patients’ practical problems have been solved is also scored lower by healthcare professionals.

The input from the open question about the main concerns patients reported and those observed and reported by professionals are presented in Supplemental Material Table 2. Completion rates of all 3 items was higher in professionals than in patients. Regarding rate of completeness for each of the items, 1a had the highest (71% for patients and 92% for professionals), item 1b followed (36% for patients and 64% for professionals) and finally, item 1c was the least responded to with 17% of patients completing it and 30% of professionals completing it. From the patient perspective, patients are more concerned with the disease itself and with their health status. Pain also occupies a relevant position, as does the concern about the impact of their health status in their family members. The concerns about their actual dependence, about their future and about death are also relevant for patients. Regarding the perspective of healthcare professionals, they much more recognise patients’ concerns about the future, as well as about their disease and health status, and about treatments they will be submitted to.

### Reliability

Table 3 shows the test-retest reliability scores for both patient and professionals’ versions, as well as the internal consistency of the three main dimensions and the global score of IPOS.

**Table 3:** IPOS reliability for patients and professionals

		Patients		Staff	
		ICC	Alpha	ICC	Alpha
*Physical symptoms*			0.725		0.660
	Pain	0.884		0.862	
	Shortness of breath	0.810		0.925	
	Lack of energy	0.851		0.713	
	Nausea	0.812		0.759	
	Feeling sick	0.794		0.644	
	Poor appetite	0.950		0.821	
	Constipated	0.938		0.706	
	Wounds in mouth or mouth dry	0.895		0.696	
	Sleepiness	0.882		0.632	
	Lack of mobility	0.887		0.852	
*Emotional symptoms*			0.615		0.658
	Anxiety	0.909		0.525	
	Family/friends worry	0.871		0.456	
	Depression	0.899		0.625	
	Feeling at peace	0.894		0.453	
*Communications/* *practical issues*			0.223		0.617
	Sharing feelings	0.810		0.514	
	Info needs	0.860		0.720	
	Practical problems	0.938		0.598	
*All dimensions*	All items		0.657		0.705

IPOS items showed good test-retest reliability (H_1_), with ICC from 0.794 (feeling sick) to 0.950 (poor appetite) for patients, and from 0.456 (family/friends worry) to 0.925 (shortness of breath) for professionals.

The Cronbach’s α measuring the internal consistency is, in general, moderate to high (H_2_) with scores of 0.657 for patients and 0.705 for healthcare professionals. All dimensions had also acceptable internal consistency, except for the dimension ‘communication/practical issues’ for patients that showed a very weak score.

### Validity

Having the content validity assured by the interviews with experts (patients and professionals) during the cultural adaptation of both versions, we started by testing construct validity. We used some sociodemographic and clinical variables and studied the behaviour of the different IPOS dimensions for different levels of those variables, regarding the patient version, for which the mean scores are presented in Table 4.

**Table 4:** Construct validity for patient and professionals looking at physical, emotional and practical issues dimensions

Variable	Value	IPOS Patient Dimensions						IPOS Professional Dimensions					
		Physical symptoms		Emotional symptoms		Practical Issues		Physical symptoms		Emotional symptoms		Practical Issues	
		Mean	Sig	Mean	Sig	Mean	Sig	Mean	Sig	Mean	Sig	Mean	Sig
Age	< 65 yo ≥ 65 yo	28.8 28.6	0.947	51.8 53.4	0.651	74.0 73.8	0.963	21.6 24.5	0.177	49.8 51.3	0.651	63.8 66.5	0.456
Gender	Male Female	26.6 31.6	0.040	50.4 55.7	0.127	71.6 76.7	0.104	22.3 24.5	0.320	50.2 51.3	0.748	63.0 68.5	0.127
Education	≤ 4 years > 4 years	28.8 28.5	0.903	52.8 52.5	0.918	75.2 71.6	0.279	24.3 21.3	0.173	50.7 50.6	0.984	64.9 66.2	0.722
Place of care	Primary care Hospital Palliative care	25.7 23.9 31.2	0.038	50.4 47.0 55.2	0.155	66.7 82.3 73.8	**0.008**	21.1 18.9 25.4	0.042	56.1 47.7 49.7	0.181	56.4 68.3 67.4	0.029
Cancer diagnosis	Yes No	28.7 28.6	0.979	53.2 50.5	0.528	75.5 67.0	**0.034**	23.4 22.8	0.841	49.7 54.9	0.186	67.5 56.4	0.012
Life expectancy	< 6 mo 6 mo – 1 yr > 1 yr	35.4 25.6 24.4	**< 0.001**	59.0 49.5 48.9	0.021	73.6 76.6 71.0	0.377	30.3 20.9 17.6	**< 0.001**	56.3 46.8 48.4	**0.022**	63.7 69.1 63.0	0.306
Phase of illness	Stable Unstable Deteriorating	25.3 27.8 34.5	0.004	49.1 54.2 57.0	0.117	75.8 73.2 71.5	0.495	19.3 23.4 29.1	**< 0.001**	44.4 53.2 58.4	**< 0.001**	68.8 60.9 65.4	0–140

Of the three sociodemographic variables studied, IPOS could only discriminate emotional symptoms based on gender (H_3_). Females showed more physical symptoms than male patients did. Regarding clinical variables, communication and practical issues have different significant scores regarding the various places of care(H_6_), meaning that higher scores correspond to inpatients and lower scores to primary care. IPOS could discriminate practical issues, based on cancer diagnosis(H_7_), physical and emotional symptoms based on life expectancy(H_8_) both for patient and professional dimensions. IPOS could discriminate physical and emotional symptoms based on phase of illness(H_9_), for professional dimensions, and physical symptoms from patients’ viewpoint. In summary, cancer patients showed more easiness in solving practical issues, patients with less than 6 months of life expectancy reported higher physical and emotional symptoms and were equally perceived as such by staff, and these were able to better recognise physical and emotional symptoms among those patients who were in a more deteriorating phase of their illness.

Regarding criterion validity, Table 5 shows there were good correlations between EQ-5D-3L index and IPOS physical and emotional symptoms and a good correlation between EQ-VAS and all IPOS dimensions. This measure correlates poorly with practical issues (H_10_).

**Table 5:** Criterion validity (Correlations)

Measure	Indicator	Physical symptoms	Emotional symptoms	Practical Issues
EQ-5D-3L	Index	−0.529 (p < 0.001)	−0.395 (p < 0.001)	0.028 (*p* = 0.748)
	EQ-VAS	−0.309 (*p* < 0.001)	−0.356 (*p* < 0.001)	0.221 (*p* = 0.010)
EORTC	Functional index	−0.532 (*p* < 0.001)	− 0.500 (*p* < 0.001)	0.156 (*p* = 0.071)
	Symptoms scale	0.659 (p < 0.001)	0.371 (p < 0.001)	−0.220 (p = 0.010)
	Quality of Life	−0.399 (*p* < 0.001)	− 0.358 (*p* < 0.001)	0.020 (*p* = 0.822)
HADS	Anxiety	−0.155 (*p* = 0.072)	− 0.443 (*p* < 0.001)	0.243 (*p* = 0.005)
	Depression	0.226 (p = 0.008)	0.308 (*p* < 0.001)	−0.088 (*p* = 0.312)

In relation to the EORTC (H_11_) the functional index showed significant correlations with both IPOS physical and emotional symptoms, the symptoms scale correlated stronger with physical and emotional symptoms and moderately with practical issues, and the quality of life measure correlated significantly with physical symptoms and with emotional symptoms.

Finally, the HADS (H_12_) correlated poorly with physical symptoms and significantly with emotional symptoms. The anxiety indicator also correlated significantly with IPOS practical issues.

## Discussion

The Portuguese IPOS is a reliable and valid measure, appropriate to use with patients with advanced, incurable illnesses. Regarding the translation process and cultural adaptation there were, as expected, adjustments made to the direct translation of some concepts and in the overall appearance of the measure. These changes are well aligned with the literature, mainly with the French [18] and Swedish [19] translations and cultural adaptions of this measure. Regarding the open question items 1a, 1b and 1c, these gave insights to some of the issues worrying patients, which was important and informative for healthcare professionals to act upon in real time. This item could also provide a good starter for the clinical appointment.

Regarding psychometric properties, there was good agreement between patient and professional ratings, especially in most physical symptoms. It is expected that some proxy ratings are lower for professionals and higher when done by proxy family ratings and indeed, there were lower proxy ratings for some items. This measure also discriminates well between life expectancy (surprise question) and phase of illness. IPOS is comparable with other measures in the field, as it showed moderate correlations with the EORTC-QLQ-C30, the HADS and the EQ-5D-3L.

These results show commonalities with validity of the original English and German IPOS versions [1] and the Japanese version [20]. Indeed, the original measures showed similar results for most psychometric properties and it is noted that the moderate correlations are expected given that IPOS measures how a person is affected by their symptoms rather than the severity of symptoms.

### Strengths and limitations

This was a multicentre study in different regions of the country and in different settings within all three sectors of the Portuguese healthcare system, thus securing good heterogeneity of our sample. As expected, in the specialised palliative care services, we observed a higher number of patients with a life expectancy under 6 months, 42 (52.5%), and in the health primary care services most patients were expected to live over one year, 20 (80%). This shows that patients with palliative needs are present in all settings in all sectors of the healthcare system and that the IPOS is good to identify those needs in patients with advanced disease. However, there are limitations, namely, most participants were outpatients, so there is less representation of more ill patients; also, it would have been interesting to capture functional status and non-cancer diagnoses, to better understand our sample.

## Conclusions

The Portuguese IPOS is a reliable and valid measure which can be used to identify palliative needs in people with advanced diseases, whether used by the patient or by a healthcare professional (proxy). Given the potential uses of patient centred outcome measures in clinical settings and healthcare system levels (micro, meso and macro), the Portuguese IPOS is an invaluable tool to be used in clinical practice and in research.

## Supplementary Information

**Additional file 1: Table 1.** Demographics, time of completion and main opinions about IPOS by patients and healthcare professionals. **Table 2.** Open question 1a, 1b, 1c: “What have been the main concerns in the past week?”

## Data Availability

The datasets generated and/or analysed during the current study are available from the corresponding author on reasonable request.

## Abbreviations

IPOS: Integrated Palliative care Outcome Scale
COSMIN: COnsensus-based Standards for the selection of health Measurement INstruments
EQ-5D-3L: Portuguese EuroQol questionnaire
EQ-VAS: numeric analogue visual scale
EORTC QLQ-C30: European Organization for Research and Treatment of Cancer Quality of Life Questionnaire Core
HADS: Hospital Anxiety and Depression Scale
ICC: intraclass correlation coefficient
