# Supplementary material for: Validation and cultural adaptation of the Integrated Palliative care Outcome Scale (IPOS) for the Portuguese population
Source: BMC Palliat Care. 2020 Nov 24;19:178. doi: 10.1186/s12904-020-00685-z (PMC7687758; doi:10.1186/s12904-020-00685-z)
Supplement: Supplementary file 1 — Additional file 1: Table 1. Demographics, time of completion and main opinions about IPOS by patients and healthcare professionals. Table 2. Open question 1a, 1b, 1c: “What have been the main concerns in the past week?” [file 12904_2020_685_MOESM1_ESM.docx]

**Supplemental Material Table 1**. Demographics, time of completion and main opinions about IPOS by patients and healthcare professionals

| **PATIENTS (N=12)** | |
| --- | --- |
| Age in years$\bar{x}$: $\bar{x}$ (SD); Min-Max | 66 (12.3); 49-90 |
| Gender: Male N (%) | 8 (66.7) |
| Disease (oncological): N (%) | 11 (91.7) |
| Filled Open Question 1: YES N (%) | 10 (83.3) |
| Filled Open Question 2: YES N (%) | 3 (25.0) |
| Time to fill in minutes: $\bar{x}$ (SD); Min-Max | 5.1 (2.5); 1.5-9.1 |
| Filled alone YES: N (%) | 5 (41.7) |
| Main comments/opinions: | *There were no difficulties regarding the instructions  *Objective, quick to fill, important questions, useful  *No questions missing, the open question allows for registering additional problems  *Easy to understand |
| **HEALTHCARE PROFESSIONALS (N=9)** | |
| Age: $\bar{x}$ (SD); Min-Max | 42.6 (10.4); 28-56 |
| Gender: Male N (%) | 2 (22.2) |
| Professional category: N(%) | Doctor 3(33.3); Nurse 2(22.2); Psychologist 2(22.2); Social Worker 1(11.1); Internal Medicine resident 1(11.1) |
| PC advanced training: | 7 (77.8) |
| Filled Open Question 1: YES N (%) | 9 (100) |
| Filled Open Question 2: YES N (%) | 6 (66.7) |
| Main comments/opinions: | *There were no difficulties regarding the instructions  *Objective, quick to fill, important questions, useful  *No questions missing, the open question allows for registering additional problems  *Perhaps it would be useful to have one or two additional items for family issues  *”poor appetite” raised some concerns regarding the inverted interpretation |

$\bar{x}$ (SD): mean(standard deviation); Min-Max: minimum-maximum scores;

N (%): absolute (relative) frequencies

PC: palliative care

**Supplemental Material Table 2** Open question 1a, 1b, 1c: “What have been the main concerns in the past week?”

|  | | IPOS Q1a | | IPOS Q1b | | IPOS Q1c | |
| --- | --- | --- | --- | --- | --- | --- | --- |
|  |  | Patient | HP | Patient | HP | Patient | HP |
| Rate of completeness N (%) |  | 96 (71.1) | 124 (91.9) | 49 (36.3) | 87 (64.4) | 22 (17) | 41 (30.4) |
| Mean N words (min-max) |  | 4.4 (1-15) | 3.9 (1-12) | 5.2 (1-15) | 3.9 (1-11) | 3.9 (1-9) | 4 (1-16) |
| Main themes N | Pain  Tired  Intestinal problems  Issues sleeping  Dyspnea  Living alone  Other physical symptoms  Fear  Worrying about the disease  Being able to work  Being a burden  Worrying about family  Not being able to do anything  Anguish  Spiritual distress  Other | 16  3  3  3  -  2  13  5  25  1  2  6  3  -  -  14 | 24  8  1  3  5  2  28  4  15  -  2  6  -  3  -  23 | 7  3  2  -  -  -  14  3  3  -  2  8  -  3  -  9 | 6  11  4  2  4  3  18  1  17  1  2  2  9  1  -  8 | 2  5  -  -  -  -  1  1  2  -  -  5  -  -  1  5 | 5  2  -  1  -  2  9  -  4  1  1  3  3  1  -  9 |
